# Delayed Multifocal Tracheal Injury Following Thyroidectomy: A Case Report and Review of the Literature

**DOI:** 10.7759/cureus.8164

**Published:** 2020-05-16

**Authors:** Matthew Shew, Christopher Boyd, Shannon Kraft

**Affiliations:** 1 Otolaryngology - Head and Neck Surgery, Washington University School of Medicine, St. Louis, USA; 2 Otolaryngology - Head and Neck Surgery, University of Kansas School of Medicine, Kansas City, USA

**Keywords:** subcutaneous emphysema, thyroidectomy, airway reconstruction, complications, tracheal injury

## Abstract

Delayed presentation of tracheal injury after thyroidectomy is a rare complication. We present the case of a 24-year-old male presenting with findings of tracheal injury 12 days after total thyroidectomy. Upon surgical exploration, multifocal, transmural tracheal injuries were identified. Repair was performed with a combination of acellular dermal matrix allograft, local-regional flaps, silicone stenting, and tracheostomy. Herein we also review published cases of delayed tracheal injury. Our findings suggest that delayed tracheal necrosis and rupture is an uncommon yet potentially devastating complication of thyroidectomy. Surgeons should maintain a low threshold to suspect such injuries when patients present with neck swelling and subcutaneous emphysema, even up to 40 days post-operatively. Complex injuries may require a multidisciplinary approach and an armamentarium of reconstructive techniques.

## Introduction

Thyroidectomy is a common procedure frequently performed for both oncologic and non-oncologic purposes. Thyroidectomy is a relatively low-risk surgery, and tracheal injury is an extremely rare complication of thyroidectomy. Typically, tracheal injuries are immediately recognized and repaired intraoperatively with little patient morbidity [1]. Delayed rupture secondary to tracheal necrosis is even less common and can present a month or more after surgery [2-5]. Discussions regarding delayed tracheal injury after thyroidectomy in the literature are limited to a handful case reports, without a clear consensus on the mechanism of injury. We review a unique case of delayed, multifocal injury to the trachea following thyroidectomy as well as its subsequent management. We also review the currently published cases of delayed tracheal injury in the literature and discuss the reported timing, presentation, and management of such injuries. While prior reports have presented cases of unifocal delayed tracheal injury, to our knowledge this is the first report of a multifocal delayed tracheal injury after thyroidectomy. The multifocal nature of this injury required additional treatment considerations and also offers additional insight into possible mechanisms of delayed tracheal injury after thyroidectomy.

## Case presentation

A 24-year-old male with metastatic papillary thyroid cancer underwent total thyroidectomy with bilateral neck dissections. The left recurrent laryngeal nerve was sacrificed due to tumor involvement. Following the procedure, the patient had to be re-intubated in the operating room due to stridor and respiratory distress. He was extubated on post-operative day (POD) 1. The otolaryngology service was consulted to evaluate the vocal folds due to known recurrent laryngeal nerve sacrifice. The patient underwent vocal fold injection augmentation on POD 5 and was discharged home on POD 7. At the time of vocal cord injection, granulation tissue was incidentally noted along the anterior tracheal wall, extending inferiorly approximately 5 mm. This was suspected to be secondary to recent intubation. Biopsy of the granulation tissue was obtained, and histopathology was later reported as consistent with "fibrulopurulent exudate and few reactive squamous epithelial cells".

**Figure 1:**
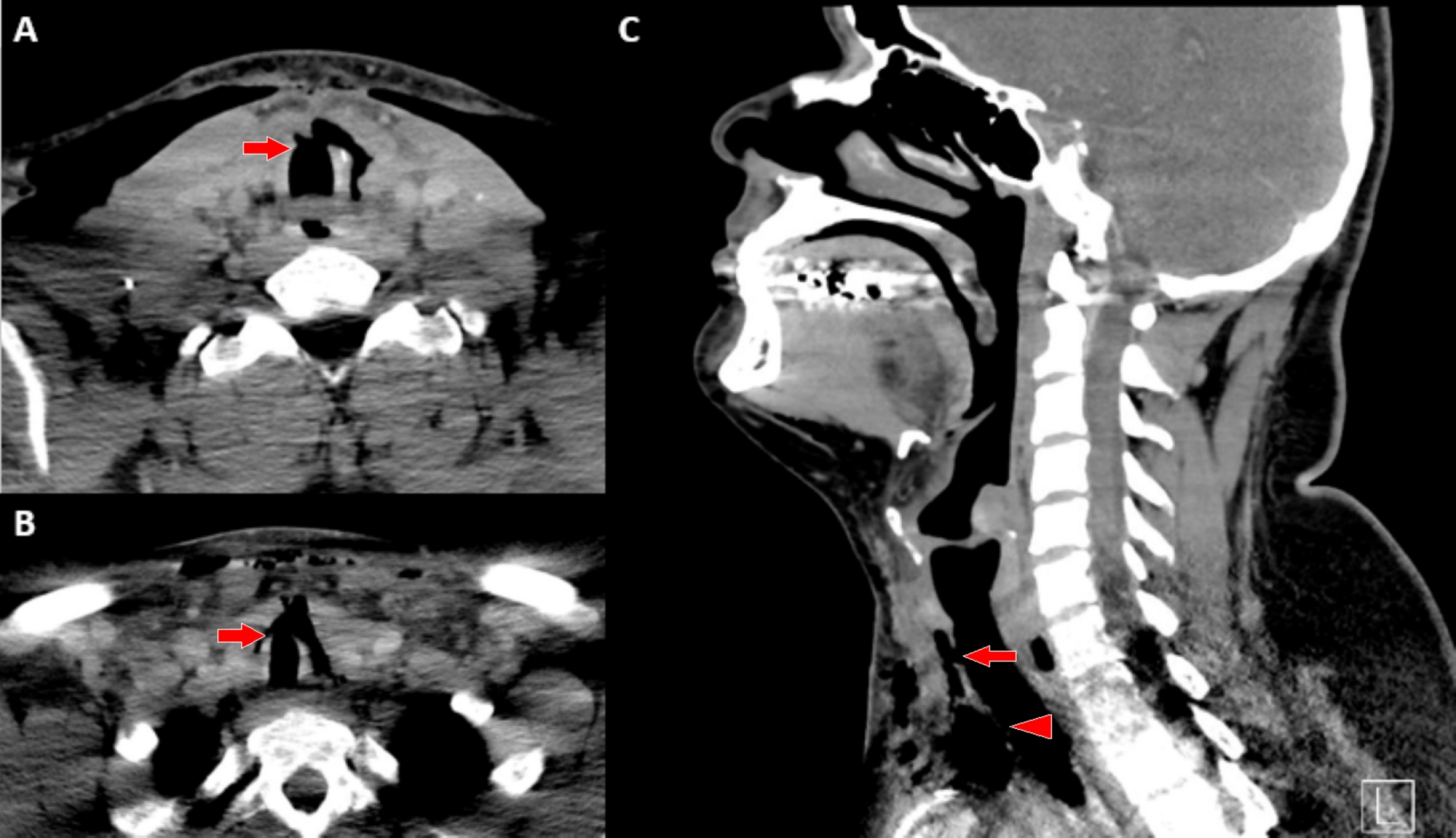
CT of the patient presenting with delayed tracheal injury CT of the neck without contrast demonstrating anterior cervical and mid-tracheal defects with extensive mediastinal and subcutaneous emphysema. (A) Axial cut of anterior cervical defect at rings 2 and 3 (arrow). (B) Axial cut of mid-tracheal defects spanning tracheal rings 9 through 11 (arrow). (C) Sagittal cut demonstrating both anterior cervical (arrow) and mid-tracheal (arrowhead) defects. CT, computed tomography

**Figure 2:**
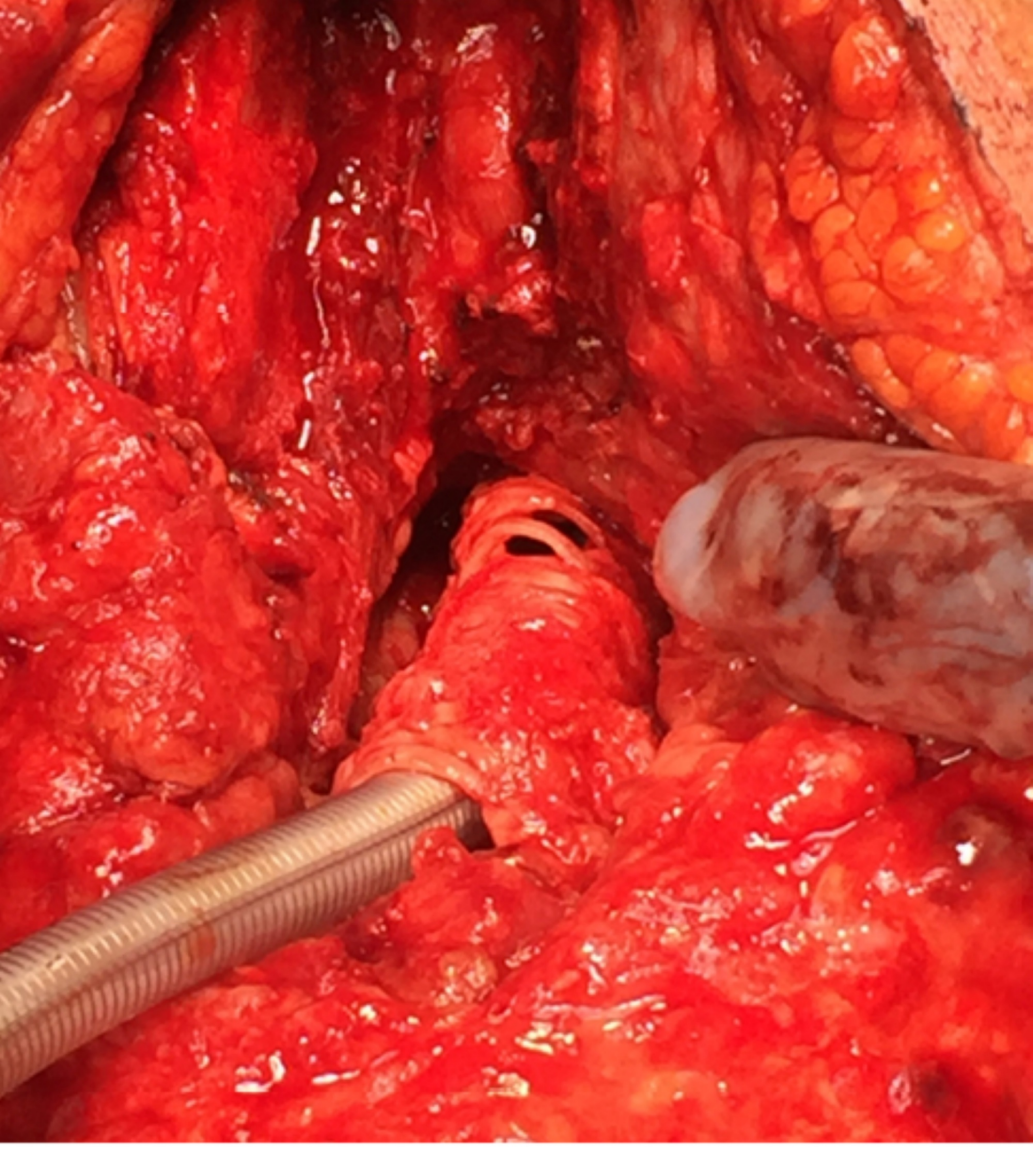
Intraoperative findings of neck exploration in the patient presenting with delayed tracheal injury Intraoperative photograph of the multifocal tracheal defects and necrosis with sparing of the posterior membranous tracheal wall. An endotracheal tube is inserted through the tracheal defect at rings 2 and 3. Partial sternotomy demonstrates tracheal defect spanning rings 9 through 11.

The patient returned to the clinic for his scheduled follow-up on POD 12. He reported to his surgeon that, for the previous one to two days, his neck would swell with cough or valsalva. CT of the neck demonstrated ventral cervical and mid tracheal defects with extensive mediastinal and subcutaneous emphysema (Figures 1A-1C). The otolaryngology service was again consulted, and the patient was taken urgently to the operating room. Intraoperatively, the following two distinct areas of tracheal injury were identified: (a) a 1-cm defect of the anterior wall at rings 2 and 3 and (b) a second defect involving perforation of the anterior intercartilaginous membranes from rings 9 to 12 (Figure 2).

**Figure 3:**
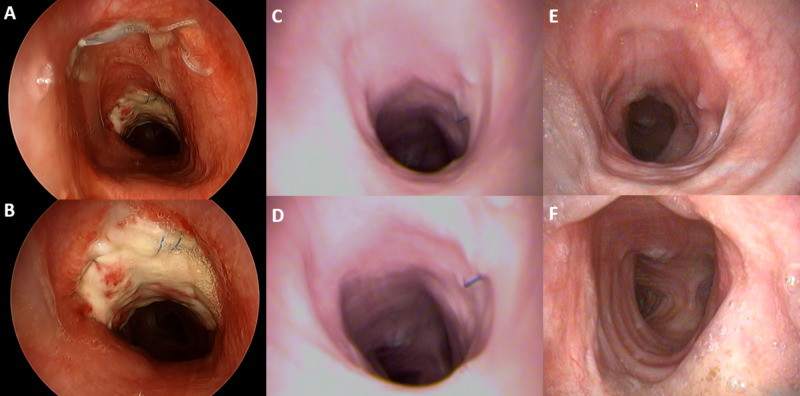
Serial bronchoscopy findings after tracheal reconstruction At three weeks (A and B) post-operatively, the allograft demonstrates good take and healing along the anterior and lateral tracheal defects. At 6 (C and D) and 12 (E and F) months follow-up, the tracheal grafts show 100% mucosalization and graft take. There is evidence of mild stenosis but good structural integrity without malacia.

A combined approach by the otolaryngology and cardiothoracic services was required to fully expose the defect. The superior defect was repaired with sternocleidomastoid flap and the inferior defect with a pectoralis flap. Due to evolution of the tracheal injury, the inferior repair had to be revised approximately two weeks later. Additional necrotic trachea was debrided. The defect was closed with a thick acellular dermal matrix allograft over a silicone stent. This was reinforced with an omental flap. The stent was removed after one week, and a tracheotomy was placed through the previously repaired proximal defect. A 7.0-mm diameter adjustable length tracheostomy tube was used to span the reconstructed trachea, functioning as both airway and stent. Three weeks post-operatively, bronchoscopy showed a patent airway with signs of epithelialization (Figures 3A,3B). Serial bronchoscopies at 6 and 12 months post-operatively revealed that the trachea was healed, with no malacia on deep respiration (Figures 3C-3F).

## Discussion

Iatrogenic tracheal injuries are well described in the literature and are most commonly a result of direct trauma during intubation. A retrospective review of 29 cases by Schneider et al. identified an increased risk of injury among obese and female patients. Patients were symptomatic shortly after injury, presenting with emphysema or pneumothoraces within 24 hours. In most cases, the injury was identified in the middle third of the trachea, and obvious linear tears were identified at the time of exploration [6].

Tracheal injuries during cervical or anterior mediastinal surgeries are uncommon. A review of more than 11,000 thyroid surgeries reported the incidence of tracheal injury at 0.06% [1]. The most common location of injury is at the posterior lateral cartilage-membranous junction near the ligament of berry and is believed to be secondary to direct trauma [1]. Mediastinoscopy also involves an anterior approach to the trachea and has a similarly low rate of injury. In a review of more than 2,021 mediastinoscopies, only three incidences of tracheal injury were identified [7]. When recognized intraoperatively, these injuries can be repaired with minimal morbidity [1].

Unlike direct injury, reports of delayed tracheal rupture following thyroidectomy or mediastinoscopy have been sparse and are limited to a handful of case reports [2-5,8-19]. Delayed necrosis and rupture can occur days to weeks after surgery. In the 18 reported cases in the literature (Table 1), the median time to presentation was POD 8 (interquartile range: 7-28 days). The longest time to presentation was 40 days. The most common presenting symptoms were neck swelling and subcutaneous emphysema.

**Table 1:** Delayed tracheal injury following thyroidectomy literature review Literature review of tracheal fistula following thyroidectomy including author, presentation, symptoms, and subsequent management. POD, post-operative day; SubQ, subcutaneous; MRND, modified radical neck dissection

Author	Age	Gender	Procedure	Indication	Time of presentation	Symptoms at Presentation	Surgical/endoscopic findings	Management
Alevizos et al. [10]	55	F	Total thyroidectomy	Multinodular goiter	POD 40	Neck swelling	Necrosis of the anterior tracheal wall	Surgical debridement, primary closure of the defect
Bertolaccini et al. [11]	38	F	Mediastinoscopy, sternal split required for hemostasis	Mediastinal adenopathy	POD 30	Fever, neck swelling, SubQ emphysema	5-mm anterior tracheal fistula	Sternohyoid/SCM flap and pectoralis flap
Bertolaccini et al. [3]	45	M	Total thyroidectomy	Papillary thyroid cancer	POD 4	Neck swelling, SubQ emphysema	6-mm anterolateral tracheal wall	Sternohyoid muscle flap
Chauhan et al. [12]	65	M	Total thyroidectomy	Medullary thyroid cancer	POD 7	Neck swelling, SubQ emphysema	5-mm anterior tracheal necrosis	Debridement, tracheotomy (decannulated POD 14)
Conzo et al. [13]	65	M	Total thyroidectomy	Thyroid nodules	POD 15	Neck swelling, pneumomediastinum, SubQ emphysema	1.5-mm anterolateral fibrocartilaginous wall	Conservative
Damrose and Damrose [4]	20	F	Total thyroidectomy	Grave's disease	POD 7	Neck swelling, SubQ emphysema	1 x 2 mm left anterior tracheal defect at ring 1	Primary closure
Golger et al. [9]	30	F	Total Thyroidectomy	Thyrotoxicosis	POD 8	Neck swelling, fever, cough	Necrosis of the right lateral trachea rings 2-4	Debridement, T-tube
Han et al. [14]	39	F	Right lobectomy	Thyroid nodule	POD 3	Fever, dyspnea, neck swelling	Right tracheal wall defect	Covered tracheal stent
Heavrin et al. [15]	55	F	Total thyroidectomy	Papillary thyroid cancer	POD 28	Neck swelling, cough	n/a	Drain placement
Jacqmin et al. [8]	53	F	Total thyroidectomy	Grave's disease	POD 8	Neck swelling, SubQ emphysema	Necrosis of anterior tracheal wall rings 1-4	Tracheal resection
Materazzi et al. [5]	39	F	Left trans-axillary lobectomy	Thyroid nodule	POD 38	SubQ emphysema	2-mm anterior tracheal defect	Curettage and fibrin glue
Mazeh et al. [16]	17	F	Total thyroidectomy	Grave's disease	POD 9	Neck swelling, SubQ emphysema	2.5 cm linear tear on anterior tracheal wall rings 2-4	Primary closure with sternothyroid flap
Philippe et al. [17]	56	F	Total thyroidectomy	Thyroid cancer	POD 6	Fever, cough, SubQ emphysema	Necrosis of the anterior tracheal wall rings 2-5	Debridement, T-tube
Sanna et al. [2]	17	F	Total thyroidectomy and MRND	Papillary thyroid cancer	POD 7	Dyspnea, fever, SubQ emphysema	3-cm defect of the right anterolateral tracheal wall	Sternocleidomastoid flap
To et al. [18]	62	M	Total thyroidectomy and MRND	Papillary thyroid cancer	POD 10	Neck swelling, SubQ emphysema	Necrosis of anterior tracheal wall rings 2 and 3	Debridement, tracheotomy
Windon et al. [19]	25	M	Right lobectomy	Papillary thyroid cancer	POD 27	Neck swelling	1-mm defect of the right lateral tracheal wall	Exploration, Penrose drain, pressure dressing

There exists no clear consensus on the mechanism of delayed tracheal injury, but hypothesized mechanisms favor a devascularization injury. Possible etiologies for such injury include devascularization of the trachea wall through thermal coagulation injury or delayed necrosis of devitalized residual tumor that had previously infiltrated the tracheal wall [2,13]. The blood supply to the trachea is composed of a complex network of multiple feeding vessels from the inferior thyroid to the bronchial and subclavian arteries [20]. Most of the vascular supply arises posteriorly along the esophagus and cervical muscles, which are not typically at risk by an anterior cervical approach. The anterior and lateral cartilaginous tracheal wall, however, is dependent upon an extensive submucosal plexus of intercartilaginous arteries that penetrate the soft tissue of the intervening tracheal rings [20]. In the case presented here, surgical exploration revealed a necrosis pattern suggestive of disruption of this submucosal plexus, whereas the posterior vascularization appeared to have been spared (Figure 2). This would explain not only the multifocal pattern but also the sparing of the posterior membranous trachea. Interestingly, 12 of the cases reported in the literature also identified necrotic injuries of the anterior or anterolateral tracheal wall, further supporting the hypothesis that thermal or ischemic compromise of the submucosal plexus contributes to the delayed presentation of injury.

Management of acute, direct iatrogenic trauma to the trachea is fairly straight forward; often patients do well with conservative management [18]. In more extensive injuries, resection and primary re-anastomosis or reconstruction with a myocutaneous flap are the options [3,8,11]. Multifocal, larger, and infected tracheal wounds, however, present a unique challenge. For unifocal defects, Sanna et al. and Bertolaccini et al. describe the use of rotational myocutaneous flaps, with good success for injuries < 3 cm [2,11]. Golger et al. described the use of a Montgomery T-tube overlaid with myocutaneous rotation flap to help stent defects greater than 3 cm [9]. The size and multifocal nature of the defect in this case made primary resection and anastomosis or the use of a rotational muscle flap alone untenable. Ultimately, the patient was successfully treated with a tracheostomy placed through the most proximal defect, and reconstruction with acellular dermal matrix and an omental flap over a stent to reconstruct the large distal defect (Figures 3A-3F). At 6 and 12 months after reconstruction, the patient had 100% mucosalization and graft take, with good structural integrity and no evidence of tracheomalacia.

## Conclusions

To our knowledge, this is the first report of a delayed, multifocal injury to the trachea following total thyroidectomy. The multifocal pattern and timing of presentation suggest compromise of the submucosal vascular supply of the trachea wall. For patients presenting with neck swelling and emphysema after thyroidectomy, one must maintain a low threshold to suspect a delayed tracheal injury, even greater than two weeks after surgery. Management may require a multidisciplinary approach and an armamentarium of reconstructive techniques.
